# How Does Low Socioeconomic Status Increase Blood Lead Levels in Korean Children?

**DOI:** 10.3390/ijerph15071488

**Published:** 2018-07-13

**Authors:** Eunjung Kim, Ho-jang Kwon, Mina Ha, Ji-Ae Lim, Myung Ho Lim, Seung-Jin Yoo, Ki Chung Paik

**Affiliations:** 1 The Environmental Health Center (Neurodevelopment), Dankook University Medical Center, 201Manghyang-ro, Dongnam-gu, Cheonan-si 31116, Chungnam-do, Korea; pdwellplan@gmail.com (E.K.); minaha@dku.edu (M.H.); limjiae@gmail.com (J.-A.L.);paperose@dankook.ac.kr (M.H.L.); yoolee1@gmail.com (S.-J.Y.); penshine@hanmail.net (K.C.P.); 2 Department of Preventive Medicine, College of Medicine, Dankook University, 119 Dandae-ro, Dongnam-gu, Cheonan-si 31116, Chungnam-do, Korea; 3 Department of Psychology, Dankook University College of Social Sciences, 119 Dandae-ro, Dongnam-gu, Cheonan-si 31116, Chungnam-do, Korea; 4 Department of Psychiatry, Dankook University College of Medicine, 119 Dandae-ro, Dongnam-gu, Cheonan-si31116, Chungnam-do, Korea

**Keywords:** lead, environmental exposure, socioeconomic status, children

## Abstract

Although studies have shown that a low socioeconomic status (SES) is associated with high blood lead levels (BLLs) in children, the mechanism underlying this observation is not well known. To determine how SES influences BLLs via environmental factors in Korean children, we conducted a population-based cross-sectional study of 4744 children aged 5–13 years. Questionnaires on sociodemographic information, environmental factors, and food consumption were administered to the children’s parents. BLLs in the study subjects were measured.The complete set of hypothesized associations was assessed using regression analysis and structural equation modeling. SES was associated with high BLLs. The total effects of nutritional factors, lead in the air and total length of nearby roads, and agriculture on BLLs were −0.062 (*p* < 0.001), 0.068 (*p* = 0.005), and 0.038 (*p* = 0.035), respectively. The direct effects of playing outdoors and SES on BLLs were 0.113 (*p <* 0.001) and −0.111 (*p <* 0.001), respectively. Although playing outdoors had a greater direct effect on BLLs than did SES, the total effect of SES (standardized *β* = −0.132, *p <* 0.001) was greater than that of other sources owing to indirect effects (*β* = −0.020, *p =* 0.004). A low SES was a major risk factor for elevated BLLs via environmental factors.

## 1. Introduction

The harmful health effects of lead exposure are well known [1]. The neurodevelopmental systems of children are vulnerable to lead toxicity, which causes developmental delays and behavioral disorders at low levels and seizures and in rare instances, death at very high levels [2]. Because the efficiency of intestinal absorption of lead in youths is greater than that in adults, children are more vulnerable to lead exposure [3,4]. Assessing blood lead levels (BLLs) and identifying sources of lead exposure in children are important owing to the fragility of children.

BLLs of Korean children are similar to those of German children (GerES: German Environmental Survey) and those of American and Canadian children. According to a German environmental survey, BLLs of Germans aged 6–8years (1.79 μg/dL) and those aged 9–11 years (1.56 μg/dL) 2003–2006 [5] were similar to those of Korean children (1.26 μg/dL) [6]. BLLs of American children were 0.97 μg/dL in 2005–2006 and 0.96 μg/dL in 2007–2008 according to the National Health and Nutritional Examination Survey IV [7]. The BLL of Canadian children was 0.87 μg/dL in 2007–2009 according to the Canadian Health Measures Survey cycle 1 [8]. These data show that BLLs of Korean children are generally similar to those of North American and European children, and the lead exposure pathway of Korean children is expected to be similar to that of North American and European children.

Lead has been depicted to be a “multimedia environmental pollutant” owing to multitudinous and various sources and pathways of potential exposure. Lead exposure has been extensively assessed throughout much of the world in the following sources: lead-soldered pipes that carry drinking water, lead in soil and dust, lead-contaminated air, and use of leaded gasoline [4,9,10,11,12]. By 1988, transportation emissions were no longer the greatest source of lead emitted into the atmosphere. Exposure from air sources has been greatly reduced since introducing unleaded gasoline and replacing lead water pipes with non-lead alternatives in many countries, including Korea [11,13,14]. Despite decreasing BLLs, pediatric lead poisoning remains a major environmental health problem among youths living in poverty and living in old housing [15,16].

Previous studies reported an association between high lead levels and low socioeconomic status (SES) (i.e., poverty or lack of parental education). The risk of lead exposure related to household factors is greater for schoolchildren of lower-income individuals [15,17,18,19]. Socioeconomic disparity is also a possible contributor to nutritional deficiencies, resulting in increased vulnerability to lead poisoning [20,21,22,23,24]. The previous studies reported the associations between BLL and environmental factors such as calcium, air pollution and poor quality housing, respectively. However, exposure pathways by which socioeconomic disparity leads to elevated BLL sand the relative impacts of the relationship among the environmental factors have not yet been completely elucidated.

This study aimed to examine the direct and indirect effects of SES on BLLs in a community-based sample of children through social contextual multilevel analysis and structural equation modeling (SEM). Because our aim was to understand how social influences are associated with health, we required models to explain various lead levels to analyze prominent factors and pathways. Such models may be necessary for understanding how social determinants of health function [25]. Making use of a sample of healthy children, we used SEM to determine environmental exposure pathways by which social inequalities can predict environmental risk factors, which in turn can forecast high BLLs.

## 2. Materials and Methods

### 2.1. Data Collection and Processing

**Figure 1:**
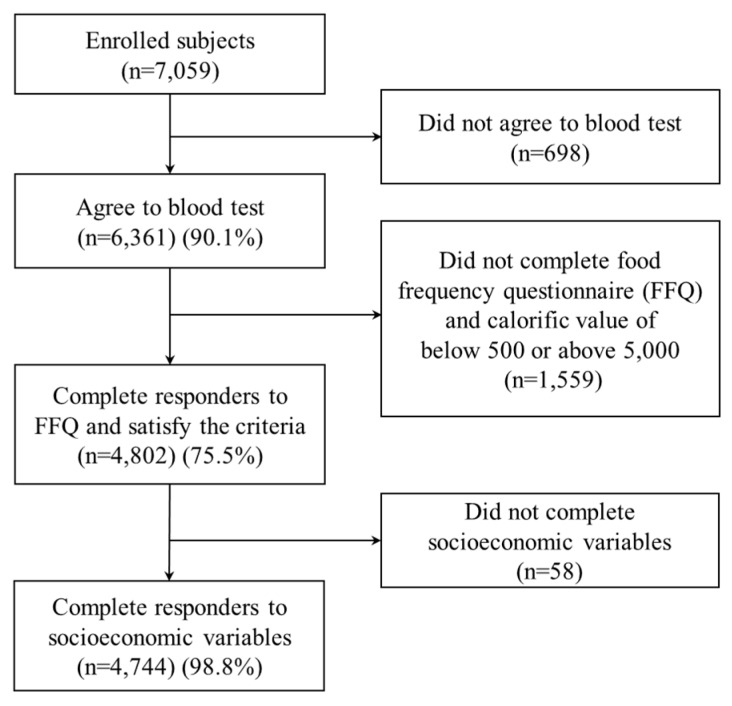
Selection of participants.

The present study, conducted by Children’s Health and Environment Research, examined children from 33 schools in 10 locations in the Republic of Korea. These schools were in urban areas (seven schools): Seoul (three schools), Daegu (two schools), and Gwangju (two schools); industrial complexes (12 schools): Incheon (three schools), Mokpo (one school), Pusan (four schools), and Yeosu (four schools); and rural areas (14 schools): Jeongeup (six schools), Cheju (three schools), and Cheonan (five schools). A quota sampling procedure was used instead of a random sampling method because it ensured that the resulting sample was a representative of the population and comprised individuals of various SES. Questionnaires on sociodemographic information, environmental factors, and dietary behavior were administered to the children’s parents. Blood samples were drawn for measuring BLLs from the children. Participants were excluded if they refused a blood test or had a daily caloric intake of <500 or >5000 kcal. The number of children who had a daily caloric intake of <500 kcal was 148 and that of >5000 kcal was 48. Completed data on BLLs, SES, and all other variables used in the analysis were available for 4744 children (67.2% of enrolled subjects) (Figure 1). The distributions of SES variables in study participants were similar with those of enrolled of subjects (Supplementary Table S1). The present study was approved by the Ethics Committee at Dankook University Medical Center. The parents/guardians provided written consent for participation. The details of the research design have been reported elsewhere [26].

### 2.2. Measures

#### 2.2.1. BLLs

Blood samples were obtained from 4744 children with special care by vein puncture using clean syringes and needles; the samples were collected in heparinized pretreated clean polypropylene tubes. All blood lead analyses were performed using the Varian SpectrAA-300 atomic absorption spectrophotometer (Varian Techtron Pty, Limited, Mulgrave, Victoria, Australia) and a graphite furnace and Zeeman background correction at a commercial laboratory. The National Institute of Standards and Technology’s Standard Reference Material 966 Toxic Metals in bovine blood was used as a standard reference and was used to evaluate the accuracy [27]. Samples were analyzed with a coefficient of variance of <3%. In our study, the limit of detection (LOD) was 0.03 μg/dL and the number of children below the LOD was two.

#### 2.2.2. SES

The socioeconomic data included paternal and maternal education and household income. Paternal and maternal education levels were classified as no school, lower school (<6 years), elementary school graduate or some middle school (6–8 years), middle school graduate or some high school (9–11 years), high school graduate (12 years), some college or college graduate (13–14 years), some university (15 years), university graduate (16 years), and graduate school or more. The average monthly household income was divided into five groups, i.e., <$1000, $1000–<$2000, $2000–<$3000, $3000–<$5000, and ≥$5000. These data were obtained from responses in the parents’ questionnaires and were used as a latent variable of socioeconomic position. SES was used to integrate paternal and maternal education and household income, and SES was divided into four categories (i.e., low, middle-low, middle, and high groups) by using cluster analysis.

#### 2.2.3. Lead in the Air and Total Length of Every Road within a 200-m Radius of the House

The lead-in-air data were the heavy metal monitoring network data of the air pollution monitoring network submitted to the National Institute of Environmental Research under the Ministry of Environment as measured by the local government institute of health and environment. The data were the monthly average of the arithmetic mean measured for the five days of the second week of every month. The air pollution concentration of individual children was calculated using IDW (Inverse distance weighted) interpolation based on the children’s home address.

Road-related data were provided by the National Road Network Map at a scale of 1:25,000 from the Korean National Traffic Database Center. Circular buffer zones with 200-m radius were constructed around each subject’s residential address using ArcGIS and the total lengths of the roads (meters) with in the circular buffers were calculated [28].

We combined total lengths of road and lead in the air as one latent variable using structural equation modeling. In structural equation modeling, the value of the latent variable was automatically generated by adding values to continuous measurement variables.

#### 2.2.4. Agriculture and Playing outside on Weekdays

A detailed survey was completed by the parents of school-aged children. Agriculture was evaluated by two questions: “Do you follow the plow?” and “Do you spray with pesticides specific to agricultural use?” to assess the participants’ agricultural work experience and use of agricultural chemicals.

Playing outdoors on weekdays indicated exposure to pollutants in the soil and was categorized into four groups based on self-reported data. The question “How long does your child play outdoors (alley, yard, playground, etc.) on weekdays?” had four possible responses: <1h, 1h–<3h, 3h–<5h, and ≥5h.

#### 2.2.5. Dietary Assessment

We used the quantitative FFQ, which includes a list of commonly consumed food items, from the Korean National Health and Nutrition Survey. It consists of food items with nine categories for consumption frequency (≥3 times/day, 2 times/day, once/day, 5–6 times/week, 3–4 times/week, 1–2times/week, 2–3 times/month, 1 time/month, and never/rarely). Portion size in the FFQ is divided into three categories: small (half of the medium portion), medium, and large (greater than the medium portion). Among the many nutrients, four nutrients of iron, protein, calcium, and zinc were selected because the results of previous studies have shown that BLLs were associated with those nutrient intake [21,22,23,29,30,31]. Calcium, iron, protein, and zinc intake were evaluated using a database and a software program [32]. The K-means cluster analysis was conducted for identifying clusters in nutrient data, which were adjusted for total energy intake using the residual method of Willett [33]. The four nutrients were standardized respectively. We assigned them to two categories (high and low) when analyzing cluster analysis. Criterion was not defined because the two groups were automatically generated by cluster analysis.

### 2.3. Statistical Analyses

Mean values and standard deviations were calculated for the observed variables. To determine the cutoff value of environmental factors associated with BLLs, exploratory data analysis was used to make the binary category for analysis. The cutoff value of the lead-in-air, the total road length, iron, protein, calcium, and zinc was the median, the upper tertile, the 10th percentile, the 25th percentile, the 75th percentile, and 75th percentile, respectively. Chi-square tests and cluster analysis were performed. We utilized the multipath component distance metric, which outperformed the squared Euclidean distance for the K-means clustering algorithm [34]. The full set of hypothesized associations was tested using a generalized linear model and SEM. The single multivariable model was used to determine the relationships between variables. We performed SEM using a two-step approach. Our study used confirmatory factor analysis of new measurement models for reliability and validity [35] and path analysis of the hypothesized conceptual model. The model included six latent variables comprising SES, lead in air and total length of roads, playing outside, nutrition, agriculture, and BLL. In the measurement model, the construct reliability values were examined by assessing the proportion of standardized coefficient and variance accounted for by each indicator. The average variance extracted (AVE) values and multiple covariates were measured to assess the discriminant validity estimates of the latent variables [36]. We use maximum likelihood with missing values imputation to address incomplete data. The analyses were performed using STATA12.

## 3. Results

### 3.1. Descriptive Statistics and Generalized Linear Models

The sex distribution of children was approximately equal (male, 51.1%; female, 48.9%). The average age of the participants was 8.23 years (standard deviation [SD], 1.3; range, 5–13). The BLLs ranged from 0.03 to 26.49 μg/dL, with an arithmetic mean of 1.78 μg/dL (SD, 0.96) and a median of 1.63 μg/dL. Overall, the children’s BLLs of our study were relatively low. The CDC′s action level of >5 μg/dl accounted for only 0.93% of the children (44(n)/4744(N)) in the current study. All children had BLLs of <10 μg/dL with the exception of one person. And BLL in children followed a normal distribution. Lead levels in the air ranged from 0.02 to 0.11 μg/m^3^ (mean, 0.06; SD, 0.02). The mean BLL in the low paternal education group (2.09 μg/dL) was greater than that in the high paternal education group (1.71 μg/dL) (*p* < 0.0001). The effect of maternal education was similar to that of paternal education. The mean BLL for the lowest household income group (1.90 μg/dL) was greater than that of the highest (1.68 μg/dL) (*p* = 0.0001). The mean BLL for the low nutrition group (1.83 μg/dL) was greater than of the high nutrition group (1.72 μg/dL) (*p* < 0.0001).Also, low iron, protein, zinc, and calcium intake were significantly associated with higher BLLs (Table 1).

Comparison of the BLLs in the low SES group with those in the higher SES groups showed that the proportion of high BLLs of ≥2 μg/dL, where 2 μg/dL is an integer value close to the 75th percentile value of BLLs (2.19 μg/dL) in our study participants, was highest in the low SES group (*p* < 0.0001).In the generalized linear models, lower SES (β = 0.224, *p* < 0.0001, p for trend = 0.001), over five hours of playing outside on weekdays (β = 0.719, *p* < 0.0001, p for trend = 0.012), high levels of lead in the air (β = 0.110, *p* < 0.0001), farmer’s children (β = 0.171, *p* < 0.0001), and lower nutritional status (β = 0.098, *p* = 0.001), boys (β = 0.201, *p* < 0.0001) and younger children (β = −0.128, *p* < 0.0001) were associated with higher BLLs (Table 2).

### 3.2. Measurement Modeling

The model fit the data well: χ^2^ (df = 74, *n* = 4744) = 569.708, comparative fit index (CFI) = 0.960, Tucker–Lewis Index (TLI) = 0.950, and root mean square error of approximation (RMSEA) = 0.038. SES consisted of paternal education (factor loading, λ = 0.826), maternal education (λ = 0.778), and household income (λ = 0.451). Lead in the air and total length of nearby roads consisted of lead in the air (λ = 0.308) and total length of every road within a 200-m radius of the house (λ = 0.861). Nutrition comprised iron (λ = 0.586), calcium (λ = 0.465), protein (λ = 0.841), and zinc (λ = 0.410). Agriculture consisted of working in agriculture (λ = 0.917) and using agricultural pesticides (λ = 0.908). The construct reliability values were 0.991 for SES, 0.918 for lead in the air and total length of nearby roads, 0.989 for nutrition, and 0.990 for agriculture. The AVE estimates were 0.975 for SES, 0.873 for lead in the air and total length of nearby roads, 0.960 for nutrition, and 0.980 for agriculture. The AVE values for latent variables were greater than the multi-covariates. Thus, convergent and discriminant validity were satisfied.

### 3.3. SEM: Blood Lead Exposure Pathways

**Figure 2:**
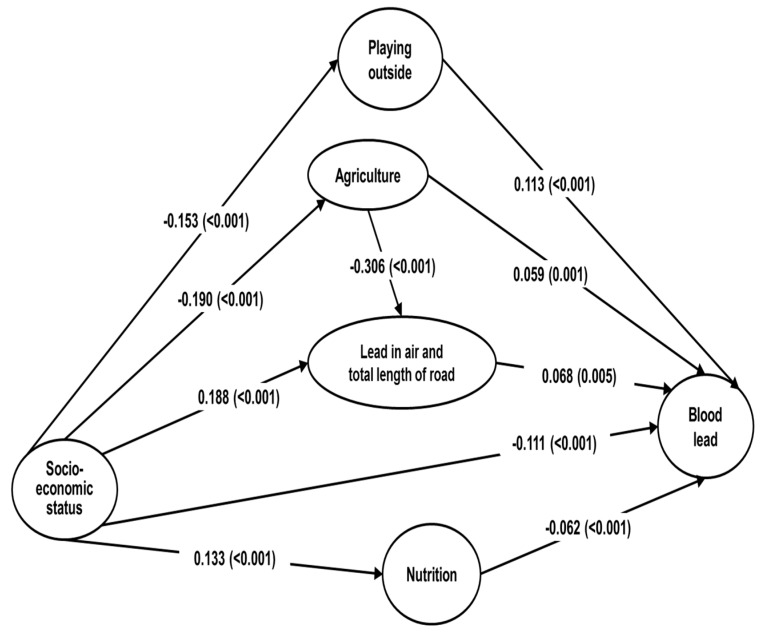
Pathways of lead exposure in a sample of Korean children defined using structural equation analysis. Footnote: all beta coefficients are standard estimates and the numbers in parentheses are *p*-values.

The model fit the data well: χ^2^ (df = 78, *n* = 4744) = 10,904.9, CFI = 0.999, RMSEA = 0.001 and Standardized RMR = 0.027. The complete model with standardized beta coefficients is presented. SES had an indirect effect (standardized β = −0.020, *p =* 0.004) and a direct effect (β = −0.111, *p <* 0.001) on BLL. SES (β = −0.132, *p <* 0.001) and nutrition (β = −0.062, *p <* 0.001) had negative total effects on BLL. Playing outside (β = 0.113, *p <* 0.001), lead in the air and total length of nearby roads (β = 0.068, *p* = 0.005), and agriculture (β = 0.038, *p* = 0.035) showed a positive total effect on BLL. SES had a negative total effect on playing outdoors (β = −0.153, *p <* 0.001), and agriculture (β = −0.190, *p <* 0.001). SES had a positive total effect on lead in the air and total length of nearby roads (β = 0.246, *p <* 0.001) and nutrition (β = 0.133, *p <* 0.001) (Table 3, Figure 2).

## 4. Discussion

The present study estimated the direct and indirect effects of SES on BLLs by pathway analysis. SEM suggested that low SES is a major risk factor for high BLLs and that low SES is indirectly affected by other sources. Although the absolute value of the direct effect of playing outdoors (standardized β = 0.113, *p <* 0.001) on BLL was higher than that of SES (β = −0.111, *p <* 0.001), the total effect of SES (β = −0.132, *p <* 0.001) was greater than that of other sources, including playing outdoors, owing to indirect effects (β = −0.020, *p* = 0.004). The factor that is most important for lead exposure pathways remains unknown [10,12]. The current study demonstrated that low SES, playing outdoors, air lead emissions and total length of every road within a 200-m radius of the house, agriculture, and poor nutrition were factors that elevated children’s BLLs. These findings support the hypotheses that BLL is multi-etiological and that SES affects BLL both indirectly and directly. SES could be a major factor in elevated BLLs in children.

Low SES was associated with BLL [17]. The mean BLL was higher in the low SES group (10.19 μg/dL) than in the high SES group (3.85 μg/dL) [15]. In addition, the median BLL of the lower paternal education group (7.1 μg/dL) was significantly higher than that of the higher paternal education groups (5.9 μg/dL). Similarly, low maternal education was associated with high BLLs [18]. The higher BLLs seen in low-SES children may be due to less educated parents being unaware of the various sources of lead in the environment, subsequently affecting children’s health, leading to a lack of preventive measures at an individual level. Household risk factors and the diverse levels of hygiene in low-SES areas are likely to exacerbate exposure to lead [19].

Playing outdoors, as directly assessed, was highly correlated with BLLs (β = 0.111, *p <* 0.001) in the current study. Lead was detected in urethane tracks, low-quality waste rubber, and waste automobile tires used in playgrounds. Children playing outdoors are exposed to lead in urethane flooring materials through direct ingestion, inhalation, and dermal contact with lead [37]. The presence of lead in soil has been documented by many empirical studies that show strong associations between neighborhood soil lead level and children’s BLL [9,38]. Playing outdoors was associated with elevated BLLs in children.

High BLLs were associated with high lead emissions in the air and the total length of every road within a 200-m radius of the house (β = 0.068, *p* = 0.005). In terms of air contamination, lead emissions in the air and total length of every road within a 200-m radius of the house were the main factors contributing to lead in the atmosphere. Road transportation and heavy motor vehicle traffic that burn coal, or oil are all responsible for lead in the air [39,40,41]. In Korea, since the introduction of unleaded fuel in 1993, the level of lead in the air and children’s BLLs have decreased. However, children’s exposure to lead remains common in Korean cities. Industrial air pollution might be one cause of lead poisoning among children in Korea [13,42]. 

Agriculture was positively related to BLLs (β = 0.038, *p* = 0.035). Agriculture may increase directly BLL (β = 0.059, *p* = 0.001), as the direct effects were significantly greater than the total effects. Farmers are exposed to agricultural pesticides, which have been associated with high BLLs [43,44]. In the present study, agriculture was hypothesized to exert a negative indirect effect on BLLs via lower lead emissions in the air and the total length of every road within a 200-m radius of the house (β = −0.021, *p <* 0.001). Thus, although urban cities had greater air lead emissions and longer roads within a 200-m radius of the house, children living in rural areas associated with low SES had higher BLLs than those living in urban areas in South Korea. These findings are in disagreement with those published by Meza-Montenegro (2013) [45].The reason for this difference may be due to differences in the SES of each country’s agricultural area. However, recent study has shown that lead exposure in rural areas is high as in our study [46].

Poor nutrition, which was related to low SES, was associated with high BLLs (β = −0.062, *p <* 0.001). Poor nutrition pertaining to low SES may multiply an individual’s susceptibility to lead. Women of higher SES, who are more likely to take calcium supplements before pregnancy, are more likely to have low lead levels [23]. Mimicking calcium and conflicting with calcium-mediated cellular processes can prevent the accumulation of lead [31]. A low concentration of micronutrients in the diet induced a high degree of retention of lead in comparison to diets containing higher mineral levels. Mineral micronutrients, including calcium [23,31], protein [30], zinc [29], and iron [21,22,29] in the intestinal lumen, may compete with lead for absorption [20,21,22,24]. Some other modifier variables included in the initial theoretical model were eventually removed from the final SEM; i.e., having an indoor dog or cat, living in a house in which someone smoked, living in a house that had recently been renovated, not washing hands, and moving to a new house.

This study had several strengths. First, we applied social contextual multilevel analysis and SEM. This analysis method considered the indirect, direct, and total effects of latent variables and identified cases where there might be correlations in potential confounders [47]. Second, this study provided epidemiological evidence that assessed the effects of BLLs of <10 μg/dL on children. The power of this study was 0.99 (≥0.80) at an α level of 0.05. Thus, this study was of sufficient size to achieve adequate power. This study also monitored a large, diverse population and assessed social factors. Data were collected from children in 33 schools in 10 locations throughout South Korea using quota sampling. A class in school was represented to the exact extent that the investigator desired, which meant that various SESs and socioeconomic disparities in health were represented.

However, this study also had several limitations. First, some information was provided by a parent-reported questionnaire, which is subject to reporting bias. Second, the study did not consider all factors that might contribute to elevated BLLs in children because of the model’s parsimony and the specific purposes of explaining exposure pathways and socioeconomic disparities. Third, the study is a cross-sectional design that cannot set up a causal relationship between the outcome and the preselected risk factors.

## 5. Conclusions

The results suggest that children from a disadvantaged socioeconomic background are more sensitive to the effects of lead exposure. Playing outside, lead in air and total length of every road within a 200-m radius of the house, agriculture, and poor nutrition were also associated with elevated BLLs. These findings have important implications for evidence-based health policy decisions of reducing children’s BLLs by comparing the relative impacts on BLL and by understanding priority on public health and environmental policy making, and of reducing health inequalities of unhealthy environment of lower SES children. Future studies are required to better understand longitudinal factors that influence the relationship between environmental factors and BLLs in children.

## Figures and Tables

**Table 1:** General Characteristics and Blood Lead Levels of the Study Population.

Variables	*n* = 4744	Mean ± SD(μg/dL)	*p*-Value *	Variables	*n* = 4744	Mean ± SD(μg/dL)	*p*-Value *
Sex				Age			
Boy	2425	1.89 ± 1.06	<0.0001	<8 year	2831	1.83 ± 1.05	0.0003
Girl	2319	1.68 ± 0.83		≥8 year	1913	1.73 ± 0.81	
Paternal education				Maternal education			
<12 year	179	2.09 ± 1.11	<0.0001	<12 year	179	2.00 ± 1.06	<0.0001
12 year	1846	1.86 ± 0.91		12 year	2342	1.83 ± 1.02	
>12 year	2234	1.71 ± 0.99		>12 year	1691	1.69 ± 0.86	
Unknown	485	1.76 ± 0.91		Unknown	532	1.83 ± 0.95	
Household income (10^3^ KRW/month) ^†^				Playing outside on weekdays			
<$1000	304	1.90 ± 0.89	0.0001	<1 h	2968	1.72 ± 0.99	<0.0001
$1000–<$2000	1004	1.88 ± 1.24		1 h–<3 h	1520	1.89 ± 0.88	
$2000–<$3000	1600	1.77 ± 0.88		3 h–<5 h	131	2.14 ± 0.95	
$3000–<$5000	1357	1.74 ± 0.88		≥5 h	24	2.53 ± 1.74	
≥$5000	442	1.68 ± 0.76		Unknown	101	1.72 ± 0.79	
Unknown	37	1.80 ± 1.05					
Lead in the air				Total length of every road within a 200-m radius of the house			
<0.054μg/m^3^	2352	1.74 ± 0.98	0.001	<200 m	3257	1.77 ± 0.86	0.25
≥0.054 μg/m^3^	2392	1.83 ± 0.93		≥200 m	1340	1.80 ± 0.96	
				Unknown	147	2.11 ± 2.23	
Farmer’s children				Pesticides use in agriculture			
No	3704	1.77 ± 0.90	0.01	No	3843	1.78 ± 0.90	0.01
Yes	463	1.93 ± 1.39		Yes	334	1.99 ± 1.56	
Unknown	577	1.75 ± 0.90		Unknown	567	1.75 ± 0.90	
Nutrition (%)							
High	1752	1.72 ± 0.87	<0.0001				
Low	2992	1.83 ± 1.01					
Iron ^‡^				Protein ^‡^			
High	4272	1.77 ± 0.97	0.003	High	3558	1.76 ± 0.98	0.0002
Low	472	1.90 ± 0.83		Low	1186	1.87 ± 0.88	
Calcium ^‡^				Zinc ^‡^			
High	1186	1.73 ± 0.87	0.009	High	1183	1.72 ± 0.85	0.002
Low	3558	1.81 ± 0.99		Low	3561	1.81 ± 0.99	

* *p*-value calculated using *t*-test or ANOVA.; ^†^ 1 US equals approximately 1078.90 KRW (23 May 2018); ^‡^ The cutoff value of the lead-in-air, the total road length, iron, protein, calcium, and zinc was the median, the upper tertile, the 10th percentile, the 25th percentile, the 75th percentile, and 75th percentile, respectively.

**Table 2:** Association Between Blood Lead and Potential Lead Exposure Sources and Epidemiologic Characteristics of Children with Blood Lead Levels ≥2 μg/dL.

Variables	β	(95% CI)	*p*-Value *	*P* for Trends *	≥2 μg/dL n (%)	*p*-Value ^†^
Socioeconomic status (%)				0.001		
High	Referent				517 (28.2)	<0.0001
Middle	0.052	(−0.03, 0.14)	0.23		194 (31.1)	
Middle-Low	0.138	(0.06, 0.21)	<0.0001		327 (34.2)	
Low	0.224	(0.14, 0.31)	<0.0001		290 (39.0)	
Playing outside on weekdays				0.012		
<1 h	Referent				850 (28.6)	<0.0001
1 h–< 3 h	0.140	(0.08, 0.20)	<0.0001		548 (36.1)	
3 h–< 5 h	0.374	(0.21, 0.54)	<0.0001		67 (51.2)	
≥5 h	0.719	(0.34, 1.10)	<0.0001		14 (58.3)	
Lead in the air						
<0.054 μg/m^3^	Referent				699 (29.7)	0.001
≥0.054 μg/m^3^	0.110	(0.06, 0.17)	<0.0001		814 (34.0)	
Farmer’s children						
No	Referent				1167 (31.5)	0.003
Yes	0.171	(0.08, 0.26)	<0.0001		178 (38.4)	
Nutrition (%)						
High	Referent				507 (28.9)	0.001
Low	0.098	(0.04, 0.15)	0.001		1006 (33.6)	
Sex						
Girl	Referent				634 (27.3)	<0.0001
Boy	0.201	(0.15, 0.25)	<0.0001		879 (36.3)	
Age						
<8 year	Referent				960 (33.9)	<0.0001
≥8 year	−0.128	(−0.19, −0.07)	<0.0001		553 (28.9)	
Intercept	1.454	(1.38, 1.53)	<0.0001			

* *p*-values and p for trends were assessed in a single multivariable model and calculated using a generalized linear model; ^†^ Results from the Chi-squared test.

**Table 3:** Direct, Indirect, and Total Effects of Potential Lead Exposure Sources on Blood Lead Levels with Standardized Parameter Estimates.

Alternative Hypothesis	Standard Coefficient	Z	*p*-Value	Acceptance and Rejection of Alternative Hypothesis
Socioeconomic status				
Blood lead (direct)	−0.111	−6.09	<0.001	Acceptance
Blood lead (indirect)	−0.020	−2.89	0.004	Acceptance
Blood lead (total)	−0.132	−7.79	<0.001	Acceptance
Playing outside				
Blood lead (direct)	0.113	7.69	<0.001	Acceptance
Nutrition				
Blood lead (direct)	−0.062	−3.72	<0.001	Acceptance
Lead in the air and total length of road				
Blood lead (direct)	0.068	2.82	0.005	Acceptance
Agriculture				
Blood lead (direct)	0.059	3.23	0.001	Acceptance
Blood lead (indirect)	−0.021	−7.72	<0.001	Acceptance
Blood lead (total)	0.038	2.11	0.035	Acceptance
Agriculture				
Lead in the air and total length of road (direct)	−0.306	−7.72	<0.001	Acceptance
Socioeconomic status				
Lead in the air and total length of road (direct)	0.188	7.11	<0.001	Acceptance
Lead in the air and total length of road (indirect)	0.058	6.28	<0.001	Acceptance
Lead in the air and total length of road (total)	0.246	8.41	<0.001	Acceptance
Socioeconomic status				
Nutrition (direct)	0.133	6.66	<0.001	Acceptance
Socioeconomic status				
Playing outside (direct)	−0.153	−8.98	<0.001	Acceptance
Socioeconomic status				
Agriculture (direct)	−0.190	−10.12	<0.001	Acceptance

## Supplementary Materials

The following are available online at http://www.mdpi.com/1660-4601/15/7/1488/s1, Table S1: Comparisons of the SES variables among the enrolled children with SES variables, the study participants, and no study participants.
