# Supplementary material for: How Does Low Socioeconomic Status Increase Blood Lead Levels in Korean Children?
Source: Int J Environ Res Public Health. 2018 Jul 13;15(7):1488. doi: 10.3390/ijerph15071488 (PMC6068902; doi:10.3390/ijerph15071488)
Supplement: Supplementary file 1 [file ijerph-15-01488-s001.pdf]

International Journal of
Environmental Research and Public Health

MDPI

Supplemental materials

Supplementary table 1. Comparisons of the SES variables among the enrolled children with SES variables, the study participants, and no study participants

	Enrolled children with SES variables		Study participants		No study participants	
Variables	N=6,729	%	N=4744	%	N=2,315	%
Paternal education						
< 12 yr	313	5.3	179	4.2	134	8.0
12 yr	2,664	44.8	1,846	43.3	818	48.7
> 12 yr	2,962	49.9	2,234	52.5	728	43.3
Maternal education						
< 12 yr	311	5.3	179	4.3	132	8.0
12 yr	3,393	57.9	2,342	55.6	1,051	63.7
> 12 yr	2,158	36.8	1,691	40.2	467	28.3
Household income (10^3 KRW/month)*						
< $1,000	536	8.1	304	6.5	232	12.1
$1,000-1,999	1,501	22.6	1,004	21.3	497	25.8
$2,000-2,999	2,196	33.1	1,600	34.0	596	30.9
$3,000-4,999	1,830	27.6	1,357	28.8	473	24.6
≥ $5,000	570	8.6	442	9.4	128	6.7

Footnote: The sum of the subcategories and the total number may be different because of the missing.
